# Cervical Spinal Osteomyelitis with Epidural Abscess following an *Escherichia coli* Urinary Tract Infection in an Immunocompetent Host

**DOI:** 10.1155/2019/5286726

**Published:** 2019-04-16

**Authors:** Abdelmoniem Moustafa, Rowida Kheireldine, Zubair Khan, Hussam Alim, Mohammad Saud Khan, Mohd Amer Alsamman, Eslam Youssef

**Affiliations:** ^1^University of Toledo Medical Center, Department of Internal Medicine, 3000 Arlington Avenue, MS 1150, Toledo, OH 43614, USA; ^2^Hospitalist Division, Miriam Hospital, Providence, RI, USA; ^3^University of Toledo Medical Center, Department of Radiology, 3000 Arlington Avenue, MS 1150, Toledo, OH 43614, USA

## Abstract

Spinal epidural abscess (SEA) is uncommon with an incidence reported as 0.33–1.96 abscesses per 10,000 hospital admissions per year. Two-thirds of the cases were caused by *Staphylococcus aureus*. *Escherichia coli* (*E. coli*) is a less common cause of SEA, and it is usually after urinary tract infection in patient with preexisting risk factor. A 69-year-old male with a past medical history significant for prostatitis was admitted with fever, altered mental status, neck pain, progressive lower extremities weakness, and frequent falls for 7 days. Both blood and urine cultures grew *E. coli*. Lumbar puncture showed 94 RBCs, 24 WBCs (16% neutrophils and 46% lymphocytes), and elevated protein level at 1140 mg/dl with no bacteria. C-spine MRI showed epidural abscess along the anterior and right lateral margin of the cord causing cord compression from C5 through C7, anterior perivertebral abscess from C4 through T2, marrow edema involving C6 and C7 vertebral bodies with increased signal in the intervertebral disc space at C6-C7, and consistent with osteomyelitis and discitis. Anterior cervical decompression with evacuation of anterior epidural abscess with fusion was done. The culture from the epidural abscess grew *E coli*. A diagnosis of SEA should be considered in patients presenting with progressive weakness and neurological deficits following UTI and is to be confirmed by MRI. *E. coli* could be the culprit for epidural abscess and spine osteomyelitis even in immunocompetent patients.

## 1. Introduction

Spinal epidural abscess (SEA) is an uncommon entity with a reported incidence of 0.33–1.96 abscesses per 10,000 hospital admissions per year [1]. The leading bacterial pathogen causing SEA is *Staphylococcus aureus*, which accounts for about two-thirds of cases [2]. *Escherichia coli* is a less common cause of SEA and is usually secondary to urinary tract infection [3]. These patients usually have preexisting risk factors such as diabetes, obesity, alcoholism, trauma, and bone degeneration. We present a case of a 69-year-old immunocompetent male patient who developed cervical spine osteomyelitis and epidural abscess caused by *E. coli* secondary to urinary tract infection.

## 2. Case Summary

A 69-year-old male with a past medical history significant for prostatitis was admitted with fever, altered mental status, neck pain, progressive lower extremities weakness, and frequent falls for 7 days. On admission, his physical examination revealed that nuchal rigidity, Kerning's signs, and Brudzinski's signs were positive. On examination of his lower extremities, spasticity was positive, and power was decreased 3/5 in both lower extremities. Knee and ankle reflexes were brisk, and the Babinski sign was present bilaterally. Fundoscopic examination was also done, and there was no papilledema. Rest of the physical examination including auscultation of the precordium was within normal limits. He underwent CT scan of the head followed by lumbar puncture because of the altered level of consciousness and positive signs of meningeal irritation. CT scan of the head was reported as normal.

CSF examination showed 94 RBCs and 24 WBCs (16% neutrophils and 46% lymphocytes). It also showed significantly elevated protein level at 1140 mg/dl with no bacterial or acid-fast bacilli growth. Myelin basic protein was elevated at 7.45 ng/ml. The glucose level was 66 mg/dl. His white blood cell (WBC) count on admission was 21.8 Thou/mm^3^ with 89% segmented neutrophils. Urinalysis showed trace leukocyte esterase, few WBCs, and many bacteria. C-reactive protein (CRP) and erythrocyte sedimentation rate (ESR) were elevated at 113 and 110, respectively. He was pan cultured and was started on empiric antibiotics including vancomycin, cefepime, and acyclovir for suspicion of meningioencephalitis. Both blood and urine cultures grew *E. coli*.

**Figure 1:**
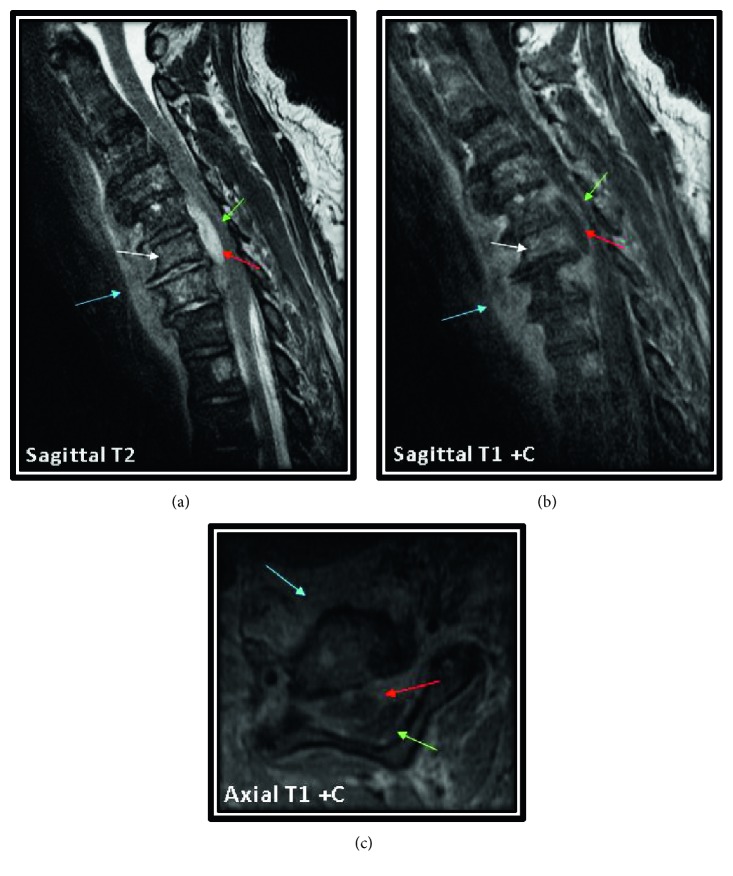
Sagittal and axial MRI scan of the cervical spine showing epidural abscess along the anterior margin of the cervical spinal cord (red arrow) causing cord compression from C5 through C7 (green arrow). There is also anterior subligamentous abscess from C4 through T2 (blue arrow) and marrow edema and enhancement involving C6 and C7 vertebral bodies (white arrow) with increased signal in the intervertebral disc space at C6-C7 consistent with osteomyelitis and discitis.

The neurology team was on board, and as per their recommendation, a magnetic resonance imaging (MRI) of the cervical spine was obtained which demonstrated epidural abscess along the anterior and right lateral margin of the cord causing cord compression from C5 through C7, anterior perivertebral abscess from C4 through T2, marrow edema involving C6 and C7 vertebral bodies with increased signal in the intervertebral disc space at C6-C7, and some enhancement of the vertebral bodies at C5 and C6 (Figure 1). These findings were consistent with osteomyelitis and discitis with epidural abscess formation. On the same day, the patient developed respiratory distress requiring intubation and mechanical ventilation. The patient was transferred to intensive care unit for further management and monitoring. Next day, anterior cervical decompression and evacuation of anterior epidural abscess with fusion were done. Postoperative fluid cultures from the epidural abscess grew *E. coli*. He was switched to IV ceftriaxone and was extubated successfully on the second postoperative day.

The patient was subsequently transferred out of the intensive care unit, and he responded well to treatment. He was actively followed by physical therapy, his power in lower extremities started improving, and he was 4/5 at the time of discharge. His inflammatory markers started trending down. His ESR went down to 24, and CRP was 18 on the 4th postoperative day. He was then discharged to rehabilitation facility for physical therapy and completion of IV ceftriaxone for 10 weeks as per decision of the infectious disease team.

## 3. Discussion

Spinal epidural abscess caused to by *E. coli* is an uncommon disease with very few cases described in the literature. The case reports describing epidural abscess caused by *E. coli* had been secondary to genitourinary conditions as urinary tract infections, pyelonephritis, prostatitis, and transrectal US prostate biopsy [3–12]. These reported cases had preexisting risk factors for epidural abscess such as diabetes, obesity, alcoholism, trauma, and bone degeneration. Three cases were reported as spontaneous SEA with no associated risk factors [6, 9, 12]. The most common presentation reported was fever with neck or back pain and tenderness. This is the first case described of a spontaneous cervical epidural abscess caused by *E. coli* following a UTI, presenting with neurological deficits in the form of progressive weakness of both lower extremities, decreased sensation in the upper extremities and bowel and urinary retention, in a previously healthy individual with no risk factors.

The leading bacterial pathogen causing SEA is *Staphylococcus aureus*, which accounts for about two-thirds of cases. Pfister et al. [13] reported *Staphylococcus aureus* as the causative organism in 63% of the cases, while *E. coli* was reported in 13% of the cases. *Escherichia coli* is a less common cause of SEA, and it is usually subsequent to urinary tract infection. In a study of 42 patients with bacterial SEA, *E. coli* was detected as the causative pathogen in two patients [14]. In another study of 39 patients with SEA, Gram-negative bacilli were found as the etiological agent in 13% of the patients [15]. Reihsaus et al. [16] reported 21 of 830 patients with SEA had positive culture for *E. coli*. Possible sources of infection reported include bone and joint infections, urosepsis, prostatic abscesses, dental abscesses, retropharyngeal abscesses, and endocarditis with most commonly reported source being skin and soft tissue infections [13, 17].

The most common sites for epidural abscess are the thoracic spine, followed by the lumbar and the cervical spine, as reported by Huang et al. Typically, the abscess involves multiple segments at the time of diagnosis. Most abscesses are posteriorly located. Anteriorly located abscesses are typically associated with vertebral osteomyelitis [17]. In our case, SEA occurred in the cervical region and was limited to two to three vertebrae.

The classical diagnostic triad of SEA consists of fever, spinal pain, and neurologic deficits [18]. However, only a small proportion of patients have all three components at presentation. A study demonstrated 71% of the patients had back/neck pain, 66% had fever, and 34% had paralysis [19]. In a case-control study, 62% reported radicular pain; 41% reported neurologic deficit, including sensory loss in 25%, subjective weakness in 35%, and difficulty with urination in 22% [20]. Four stages may be identified in SEA development: stage I: back/neck pain at the level of the affected spine, fever, and spine tenderness; stage II: radicular pain radiating from the affected part of the spinal cord; stage III: neurological deficits such as hypoesthesia, motor weakness, bowel, or bladder dysfunction; stage IV: paralysis [5]. Once paralysis develops, it may quickly become irreversible. Thus, urgent intervention is required if progression of weakness or other neurologic findings are detected. Although SEA at any level is a serious condition, it is particularly devastating in the upper cervical region due to the fragility of the atlantoaxial joint. Spinal cord compression can impact breathing, since the diaphragmatic innervation is from C3, C4, and C5 [21]. In our case, the patient presented in stage III with fever and progressive weakness in both lower extremities followed by difficulty breathing due to cord compression. Surgical decompression improved the clinical condition of the reported patient.

The initial diagnostic tests include inflammatory markers such as ESR and CRP and cultures (blood and urine), followed by MRI of the spine. In a case-control study, the erythrocyte sedimentation rate (ESR) was elevated in 98% of patients [18]. In a 9-month substudy of 86 patients presenting to the ED with spine pain due to SEA, the ESR was elevated in 100% of the patients with SEA, and the CRP was significantly elevated in 87% of the patients [20]. Blood and urine cultures should be obtained in all patients; however, in up to 41% of cases of SEA, blood cultures have been reported negative [16]. MRI is often positive early in the course of the infection and provides the best visualization of the location and extent of inflammatory changes. MRI has the greatest diagnostic accuracy with the reported predictive values include sensitivity up to 95% and specificity over 90% [16]. CT-guided biopsy is essential to isolate the etiologic organism.

Lumbar puncture for CSF examination is often not performed as the diagnostic yield is low with a risk of introducing infection. CT or MRI is mandatory prior to lumbar puncture to evaluate the location and the extent of the SEA for correct needle placement. In most cases, the CSF findings are nonspecific. Findings are suggestive of parameningeal inflammation but are not specific for epidural infection. The findings in our patient's CSF are similar to those described by other authors [22, 23]. The white blood cell count was elevated as well as highly elevated protein level with gram stain negative and cultures showing no growth. Myelin basic protein was elevated, which indicates acute myelin breakdown as a result of infection. The patient's CSF showed marked xanthochromia, with elevated albumin and IgG levels. The CSF IgG index was elevated; however, testing for oligoclonal bands was negative. These findings were out of proportion to xanthochromia.

Surgical decompression and drainage with systemic antibiotic therapy is the treatment of choice for many patients. In many cases, early surgical decompression and drainage are critical factors that will improve the ultimate prognosis. Urgent intervention may be required if acute or progressive neurologic deficits are detected. In the upper cervical spine epidural abscess, where a large untreated epidural abscess can render the patient ventilator-dependent, surgical management is crucial and should be performed as early as possible [16]. After surgery, patients in stage III may have no weakness or a lesser degree of weakness, whereas patients in stage IV may benefit from surgery only if they undergo decompression in 24–36 hours after the onset of neurological symptoms [5].

Empiric antibiotics should be started as soon as the diagnosis of SEA is strongly suspected and immediately following two sets of blood structures. Coverage for Gram-negative bacteria should be warranted particularly in the presence of documented or suspected infection, such as in the urinary tract. The appropriate duration of antimicrobial therapy is usually four to six weeks. However, it differs on a case-by-case basis according to the clinical, laboratory (WBC count, CRP, and ESR), and radiographic response to therapy. The first follow-up MRI is obtained at about four to six weeks if the patient is improving or at any time, if clinical deterioration occurs.

The reported outcomes of SEA as described by Danner and Hartman included 54% complete recovery, 23% with residual weakness, 9% paralysis, and 14% death. The outcome is significantly affected by the time from admission to specific diagnosis, with cases diagnosed early completely recovering, the location of the abscess, and the severity of the neurological impairment before treatment [14].

In conclusion, early diagnosis and intervention improves the prognosis in patients with SEA. A diagnosis of SEA should be considered in patients presenting with progressive weakness and neurological deficits following UTI and is to be confirmed by MRI. Despite the advances of diagnostic and management methods, about 30% of patients with SEA still have an unfavorable outcome [14]. Increased awareness of the disease and a high suspicion index is essential for rapid intervention.

## 4. Conclusion

A diagnosis of SEA should be considered in patients presenting with progressive weakness and neurological deficits following UTI and is to be confirmed by MRI.

## References

[1] Ptaszynski A. E., Hooten W. M., Huntoon M. A. (2007). The incidence of spontaneous epidural abscess in olmsted county from 1990 through 2000: a rare cause of spinal pain. *Pain Medicine*.

[2] Tong S. Y. C., Davis J. S., Eichenberger E., Holland T. L., Fowler V. G. (2015). *Staphylococcus aureus* infections: epidemiology, pathophysiology, clinical manifestations, and management. *Clinical Microbiology Reviews*.

[3] O’Neill S. C., Baker J. F., Ellanti P., Synnott K. (2014). Cervical epidural abscess following an *Escherichia coli* urinary tract infection. *Case Reports*.

[4] Al-Hourani K., Frost C., Mesfin A. (2015). Upper cervical epidural abscess in a patient with Parkinson disease: a case report and review. *Geriatric Orthopaedic Surgery & Rehabilitation*.

[5] Rosc-Bereza K., Arkuszewski M., Ciach-Wysocka E., Boczarska-Jedynak M. (2013). Spinal epidural abscess: common symptoms of an emergency condition. *A Case Report Neuroradiology Journal*.

[6] Kaya M., Kösemehmetoğlu K., Yildirim C. H., Orman G., Çelebi Ö, Taşdemiroğlu E. (2012). Spondylodiscitis as a spinal complication of transrectal ultrasound-guided needle biopsy of the prostate. *Spine*.

[7] Liu J.-H., Lin P.-W., Liu Y.-L., Liao P.-Y. (2007). Cervical spinal osteomyelitis with epidural abscess: a rare complication after interferon therapy following acute pyelonephritis. *Nephrology*.

[8] Wessling H., De Las Heras P. (2003). Cervicothoracolumbar spinal epidural abscess with tetraparesis: good recovery after non-surgical treatment with antibiotics and dexamethasone: case report and review of the literature. *Neurocirugia*.

[9] Dobson G., Cowie C. J. A., Holliman D. (2015). Epidural abscess with associated spondylodiscitis following prostatic biopsy. *Annals of The Royal College of Surgeons of England*.

[10] Fradet V., McCormack M., Perrotte P., Karakiewicz P., Saad F. (2005). An epidural abscess following transrectal ultrasound-guided biopsies of the prostate. *Canadian Journal of Urology*.

[11] Nussbaum E. S., Rigamonti D., Standiford H., Numaguchi Y., Wolf A. L., Robinson W. L. (1992). Spinal epidural abscess: a report of 40 cases and review. *Surgical Neurology*.

[12] Akagawa M., Kobayashi T., Miyakoshi N. (2015). Vertebral osteomyelitis and epidural abscess caused by gas gangrene presenting with complete paraplegia: a case report. *Journal of Medical Case Reports*.

[13] Pfister H.-W., Klein M., Tunkel A. R., Scheld W. M., Scheld W. M., Whitley R. J., Marra C. M. (2014). Epidural abscess. *Infections of the Central Nervous System*.

[14] Danner R. L., Hartman B. J. (1987). Update of spinal epidural abscess: 35 cases and review of the literature. *Clinical Infectious Diseases*.

[15] Krishnamohan P., Berger J. R. (2014). Spinal epidural abscess. *Current Infectious Disease Reports*.

[16] Reihsaus E., Waldbaur H., Seeling W. (2000). Spinal epidural abscess: a meta-analysis of 915 patients. *Neurosurgical Review*.

[17] Huang C. R., Lu C. H., Chuang Y. C. (2011). Clinical characteristics and therapeutic outcome of Gram-negative bacterial spinal epidural abscess in adults. *Journal of Clinical Neuroscience*.

[18] Darouiche R. O. (2006). Spinal epidural abscess. *New England Journal of Medicine*.

[19] Davis D. P., Salazar A., Chan T. C., Vilke G. M. (2011). Prospective evaluation of a clinical decision guideline to diagnose spinal epidural abscess in patients who present to the emergency department with spine pain. *Journal of Neurosurgery: Spine*.

[20] Davis D. P., Wold R. M., Patel R. J. (2004). The clinical presentation and impact of diagnostic delays on emergency department patients with spinal epidural abscess. *Journal of Emergency Medicine*.

[21] Al-Hourani K., Al-Aref R., Mesfin A. (2016). Upper cervical epidural abscess in clinical practice: diagnosis and management. *Global Spine Journal*.

[22] Sendi P., Bregenzer T., Zimmerli W. (2008). Spinal epidural abscess in clinical practice. *QJM*.

[23] Baker A. S., Ojemann R. G., Swartz M. N., Richardson E. P. (1975). Spinal epidural abscess. *New England Journal of Medicine*.

